# Supplementary material for: The Hungarian hubris syndrome
Source: PLoS One. 2022 Aug 24;17(8):e0273226. doi: 10.1371/journal.pone.0273226 (PMC9401175; doi:10.1371/journal.pone.0273226)

Supplemental Material for Magyari et al., The Hungarian hubris syndrome

Supplementary Methods

Subjects

Parliamentary elections are held every four years in Hungary; however, a PM may be replaced earlier by the ruling political party. In 1998, 2002, and 2010 elections led to defeat of the incumbent party (Figure 1), while in 2006 and 2014 the incumbent party and PM held onto power. In 2004 and 2009 PMs resigned and were replaced by their party (MSZP: Hungarian Socialist Party) and governed until the next elections. In 1998 Viktor Orbán and the conservative Fidesz (Alliance of Young Democrats) and in 2002 Péter Medgyessi and left-wing MSZP beat the incumbent and won the election. In 2004, Medgyessi resigned due to an internal conflict between the MSZP and its liberal coalition partner SZDSZ (Alliance of Free Democrats), and Ferenc Gyurcsány became PM by a parliamentary vote. In the elections of 2006, Gyurcsány secured a second term as PM, however, in 2009 he needed to resign due to a constructive motion of no-confidence against him. He was followed by Gordon Bajnai as PM of the MSZP government, until the 2010 parliamentary elections when Orbán and his Fidesz party, by that time a populist, nominally a nationalist-conservative party with intimate ideological ties to the far-right (for the political and legal changes towards an authoritarian structure in Hungary under Orbán see (Körösényi et al., 2020)).

Supplementary Results

Frequency of WE and Other-referential personal pronouns

We compared the frequency of WE (first-person plural personal pronouns) and all other pronouns (Non-WE) in the different parliamentary cycles of Orbán's and Gyurcsány's

premierships by Chi-square statistics (Figure 1 and Table S1). For Gyurcsány, there was a significant increase in the frequency of WE compared to Non-WE between his first and second premiership ($X^2(1) = 17.524$, $p < .0001$). For Orbán, there was also a significant difference in the frequency of WE and Non-WE through his premierships ($X^2(2) = 11.735$, $p = .003$). When we compared Orbán's premierships in pairs, there was an increase in the frequency of WE compared to Non-WE between the first and second premiership ($X^2(1) = 10.977$, $p = .001$), and there was no difference between the second and third premiership ($X^2(1) = 0.592$, $p = .442$).

We also compared the frequency of Other-referential pronouns compared to the frequency of all other pronouns (Non-Other) in the different parliamentary cycles of Orbán's and Gyurcsány's premierships. There was no difference in the frequency of Other-referential pronouns compared to all other pronouns between Gyurcsány's premierships ($X^2(1) = 1.177$, $p = .278$). For Orbán, there was a significant difference in the frequency of Other and Non-Other through his premierships ($X^2(2) = 6.239$, $p = .044$) which could show a decrease in the frequency in Other from his first to third premiership. When Orbán's premierships were compared in pairs, there was no difference in the frequency of Other compared to Non-Other between the first and second premiership ($X^2(1) = 0.943$, $p = .332$) and between the second and third premiership ($X^2(1) = 2.334$, $p = .127$), but there was a difference between his first and third premiership ($X^2(1) = 5.207$, $p = .022$). We also checked the differences in frequency separatly for the third-person plural personal pronouns („they"). There was no difference in the frequency of „they" compared to all other pronouns among all premierships of Orbán and Gyurcsány (Gyurcsány: $X^2(1) = 0.488$, $p = .485$; Orbán: $X^2(2) = 3.132$, $p = .209$).

Members of Parliament vs. Prime Ministers

We also tested parliamentary speeches of Orbán and Gyurcsány when they were not PMs, but regular Members of Parliament (MPs). It should be noted, however, that only a rather limited data set was available (number of speech samples < 10, see Table S6).

First, we tested whether the WE:I ratio (both for PP and for VC) decreases when a PM is voted out of power. We compared speeches in parliamentary cycles right before losing an election (Gyurcsány: 2006-2009; Orbán: 1998-2002) with speeches from the parliamentary cycle right afterwards (Orbán: 2006-2009; Gyurcsány: 2010-2014), which we labelled as parliamentary cycles. There was no difference between the PMs, nor an interaction between PMs and parliamentary cycles in WE:I ratio for PP, but a marginal difference between the two types of parliamentary cycles (Table S7). In a subsequent analysis, one outlier speech sample (n>30) was excluded to improve model diagnostics, but the pattern of results remained very similar (see Table S8-S9, Figure S5). Similarly, to the above analysis, for VC, there were only marginal differences between parliamentary cycles, but there were neither any differences between MPs nor an interaction (Table S6 and S10). An improved model showed similar results after the exclusion of two outlier speech samples (n>50) (see Table S8 and S11, Figure S6).

Before the first premiership

We also compared WE:I ratio for VC in the parliamentary cycle preceding Orbán's and Gyurcsány's first premiership with the cycle in which they were PMs for the first time (parliamentary cycles). Orbán was already a Member of Parliament between 1994 and 1998 preceding his premiership, therefore, we extended our collection of speeches for Orbán to include this period. Gyurcsány was a Minister for Children, Youth and Sports from May 19, 2003 to September 29, 2004, before he became PM. Both men participated in a number of parliamentary debates in the years preceding their first premierships. In the archive of the

Parliamentary records before 1998, the speech categorization we used to classify speeches as spontaneous as possible was not available. Therefore, we evaluated each of Orbán's speech samples between 1994 and 1998 and found that they were all replies to others during debates. This analysis was done only on WE:I ratio for VC because there was no sufficient data available for PP (speech samples < 10, items < 20). According to our analysis of VC, there was no difference between PMs but there was a difference between parliamentary cycles. The WE:I ratio for VC was higher in the parliamentary cycle before the first premiership than after (see Table S12-S13, Figure S7).

Supplementary Discussion

In the additional analysis we examined whether the first-person and third-person plural forms increased from the first to their second premiership for both Orbán and Gyurcsány. The use of the first-person plural ("we","us") and the third-person plural pronouns ("they", "them") can also set up a contrast between an ingroups and outgroups in political speech which is a prominent feature of the expression of ideologies (van Dijk, 2006). This theory would predict that we find and increase in the frequency of both first and third-person plural forms. While there was increase in the frequency of the first-person plurals (compared to all other personal pronouns) between the first and second premiership of both Orbán and Gyurcsány in our speech samples, there was no difference in the frequency of the third-person plural pronouns.

In these additional analyses we also compared parliamentary speeches of Orbán and Gyurcsány when they were in or out of office as PMs. These analyses should be interpreted with caution due to the low number of speeches made out of office (Table S6 and S12). We found that both Orbán and Gyurcsány had a higher WE:I ratio for VC in the parliamentary

cycle preceding their first premiership. We also found that WE:I ratio did not decrease for VC and PP when they were not PMs anymore.

Regarding the WE:I ratio in speeches made in parliamentary cycles following a parliamentary cycle of premiership and power, the interpretation of hubris as a syndrome (Owen & Davidson, 2009) predicts that the ratio of plurals should decrease, because the main invoking criteria of hubris, the position of power is lost. We found that this prediction was not borne out, as we found no such reduction, even though it should be noted that not detecting any change might be due to a lack of statistical power. Nevertheless, the percentage of WE is numerically higher for both politicians after they were voted out of office. It should be noted that Orbán's ratio of plurals was relatively low during his first premiership compared to his second one, therefore, it may not less surprising that his WE:I ratio did not decrease further when he was out of office. In contrast, Gyurcsány lost his position after becoming PM the second time, when he already had an increased ratio of WE:I (relative to his first premiership). Thus, it is quite surprising that the WE:I ratio did not decrease in his case. Gyurcsány lost power due to political bargains and he was forced to resign in 2009 one year before the elections, for which MSZP, his party, did not nominate him for PM. However, he remained politically active and eventually founded a new party (Democratic Coalition, DK) in 2011. The lack of decrease in the WE:I ratio may suggest that hubris may be less a temporary syndrome invoked by power positions, but a permanent personality distortion, which may or may not be restrained by later life events. Specifically, hubristic features might be still present after a leader lost power but keeps working on regaining it again. Future research could examine what are the exact conditions for a decrease of hubris when leaders get out of their powerful positions.

Regarding the parliamentary cycles preceding first premierships, we predicted that markers of hubris will be lower, as HS should be elicited by gaining high political office and

experiencing powerful and excessive success. We could not confirm this hypothesis either, because we found that the WE:I ratio for VC was higher both for Orbán and Gyurcsány before than during their first premiership. First, these politicians had been in powerful positions already before they became PMs. Gyurcsány held a Ministerial position in the parliamentary cycle preceding his premiership and was a CEO of large companies for long periods. Orbán was a president of his party before his first premiership for about a decade. Even though re-election had a strong influence on Orbán's and Gyurcsány's hubris, they might already have had a predisposition for such personality traits before they became PMs. These observations corroborate the possibility that hubris may not be as much a syndrome but an inclination or a kind of personality structure, which prompts individuals who have experienced success and leadership to enhance their power by striving ever higher on the ladder. It is in line with the idea that a less intensive from of hubris might be adaptive for gaining leadership (Asad & Sadler-Smith, 2020; Owen & Davidson, 2009; Zeitoun et al., 2019). There is a more intricate picture emerging here, however. Gaining power may have a pacifying effect initially, as the responsibilities, uncertainties and burdens of top leadership become clear. However, re-gaining power may allow for a kind of uninhibited attitude, which lets the genie of hubris out of the lamp. We suggested earlier that in Eastern-Europe it might be the case that strongmen strive for power, but that the institutional system resists less, which may seem to be in contradiction with the high WE:I ration prior to being elected in office for the first time. However, there is a clear reduction in the WE:I ratio, specifically in the first term, instead of excessive expression of hubristic features, which may be expected from a strongman, who just got what he wanted: power. It is still a question whether such a reduction in hubristic linguistic markers is a strategic suppression effect (e.g., in hope of re-election) or a genuine humbling effect of responsibility and statesmanship. Future research

could further investigate the dynamics and immediate effects of raising in the power hierarchy on hubristic traits.

Supplementary Tables

Table S1

Frequencies of Personal Pronouns in the Parliamentary Speeches of the Four Hungarian PMs

		Personal pronouns					
		1st		2nd		3rd	
		Singular[a]	Plural[a]	Singular[a]	Plural[a]	Singular[a]	Plural[a]
Prime ministers	N (words)[b]	Self[c]	«WE»[c]	Audience[c]		Other[c]	
P. Medgyessy (2002-2004)		40	18	45	25	22	9
	6901	5.796	2.608	**10.143**		4.492	
F. Gyurcsány (2004-2006)		20	6	53	11	14	1
	4018	4.978	1.493	**15.928**		3.733	
F. Gyurcsány (2006-2009)		39	45	29	21	29	5
	6296	6.194	7.147	**7.942**		5.4	
G. Bajnai (2009-2010)		26	16	17	9	18	3
	4195	**6.198**	3.814	**6.198**		5.006	
V. Orbán (1998-2002)		33	24	68	28	25	10
	7288	4.528	3.293	**13.172**		4.802	
V. Orbán (2010-2014)		88	115	142	47	33	37
	14875	5.916	9.546	**12.706**		4.706	
V. Orbán (2014-2018)		105	124	175	80	34	30
	15453	6.795	11.325	**16.502**		4.142	
Total	59026	5.947	5.896	**12.706**		4.574	

Note. Category of pronouns with highest frequency are in bold.

[a]Frequency.

[b]Number of all words.

[c]Standardized frequency (/1000 words).

Table S2

Frequencies of Verbal Conjugations in the Parliamentary Speeches of the Four Hungarian PMs After Exclusion of Outlier Speech Samples

	First-person verbal conjugations		
Prime ministers	n	%	n (s.s.)
P. Medgyessy (2002-2004)	316	30	58
F. Gyurcsány			
First (2004-2006)	115	20	31
Second (2006-2009)	118	26	15
G. Bajnai (2009-2010)	228	36	27
V. Orbán			
First (1998-2002)	379	32	53
Second (2010-2014)	730	46	99
Third (2014-2018)	954	40	137

Note. n (s.s.) = number of speech samples. % = percentage of plurals

Table S3

GLMM Results for First-Person Verb Conjugations in the Parliamentary Speeches of the Four Hungarian PMs After Exclusion of Outlier Speech Samples

Fixed effect	β	SE	z	*P*	χ^2	df	*p*
					19.01	3	.0003
Intercept: Orbán[a]	-0.265	0.121	-2.195	.0282			
Medgyessy[b]	-0.836	0.2172	-3.850	.0001			
Gyurcsány[b]	-0.884	0.341	-2.595	.0095			
Bajnai[b]	-0.538	0.258	-2.080	.0375			

Note. Response variable: verb conjugations (singular = 0, plural = 1). Significance of fixed effect was evaluated by model-comparison using likelihood ratio test: a model containing the fixed effect was compared to an intercept-only model.

[a] reference category for the different levels of the fixed effects ([b])

Table S4

GLMM results for First-Person Verbal Conjugations in Orbán's and Gyurcsány's Parliamentary Speeches During Their First and Second Premiership After Exclusion of Outlier Speech Samples

Fixed effects	β	SE	z	*P*	χ^2	df	*p*
Prime ministers[a]	-0.777	0.203	-3.825	.0001	14.703	1	.0001
Premierships[b]	0.627	0.161	3.907	<.0001	15.213	1	<.0001

Note. Response variable: verb conjugations (singular = 0, plural = 1). Significance of a fixed effect was evaluated by model-comparison using likelihood ratio test: a model containing the fixed effect was compared to a model containing both fixed effects.

[a] reference-category: Orbán. [b] reference-category: first premiership.

Table S5

GLMM results for First-Person Verbal Conjugations in Orbán's Parliamentary Speeches During his Second and Third Premiership After Exclusion of Outlier Speech Samples

Fixed effect	β	SE	z	*P*	χ^2	df	*p*
Premierships[a]	-0.267	0.142	-1.881	.06	3.522	1	.0605

Note. Response variable: verb conjugations (singular = 0, plural = 1). Significance of a fixed effect was evaluated by model-comparison using likelihood ratio test: a model containing the fixed effect was compared to an intercept-only model.

[a] reference-category: second premiership.

Table S6

Frequencies of First-Person Personal Pronouns and Verbal Conjugations in Orbán and Gyurcsány's Parliamentary Speeches as PMs and as member of parliament

	First-person personal pronouns			First-person verbal conjugations		
Prime ministers	n	%	n (s.s.)	n	%	n (s.s.)
F. Gyurcsány						
Second (2006-2009)	84	54	12	317	45	17
MP (2010 – 2014)	21	57	6	55	45	6
V. Orbán						
First (1998-2002)	57	42	26	379	32	53
MP (2006-2009)	23	65	6	94	39	6

Note. n (s.s.) = number of speech samples. MP = member of parliament

Table S7

GLMM Results for First-Person Personal Pronouns in Orbán and Gyurcsány's Parliamentary Speeches as PMs and as member of parliament

Fixed effects	β	SE	z	*P*	χ^2	df	*p*
Prime ministers[a]	-0.221	0.473	-0.468	.64	0.222	1	.6428
Parliamentary cycles[b]	-0.897	0.549	-1.635	.102	3.005	1	.083

Note. Response variable: first-person personal pronouns (singular = 0, plural = 1).

Significance of a fixed effect was evaluated by model-comparison using likelihood ratio test: a model containing the fixed effect was compared to a model containing both fixed effects.

[a] reference-category: Orbán. [b] reference-category: premiership.

Table S8

Frequencies of First-Person Personal Pronouns and Verbal Conjugations in Orbán and Gyurcsány's Parliamentary Speeches as PMs and as member of parliament After Exclusion of Outlier Speech Samples

	First-person personal pronouns			First-person verbal conjugations		
Prime ministers	n	%	n (s.s.)	n	%	n (s.s.)
F. Gyurcsány						
Second (2006-2009)	53	53	11	118	26	15
MP (2010 – 2014)	21	57	6	55	45	6
V. Orbán						
First (1998-2002)	57	42	26	379	32	53
MP (2006-2009)	23	65	6	94	39	6

Note. n (s.s.) = number of speech samples. % = percentage of plurals. MP = member of parliament

Table S9

GLMM Results for First-Person Personal Pronouns in Orbán and Gyurcsány's Parliamentary Speeches as PMs and as Member of Parliament After Exclusion of Outlier Speech Samples

Fixed effects	*β*	SE	z	*P*	χ^2	df	*P*
Prime ministers[a]	-0.142	0.527	-0.269	.7876	0.072	1	.7884
Parliamentary cycles[b]	-1.008	0.591	-1.707	.0878	3.222	1	.0727

Note. Response variable: first-person personal pronouns (singular = 0, plural = 1). Significance of a fixed effect was evaluated by model-comparison using likelihood ratio test: a model containing the fixed effect was compared to a model containing both fixed effects.

[a] reference-category: Orbán. [b] reference-category: premiership.

Table S10

GLMM Results for First-Person Verb Conjugations in Orbán and Gyurcsány's Parliamentary Speeches as PMs and as Member of Parliament

Fixed effects	β	SE	z	*P*	χ^2	df	*P*
Prime ministers[a]	-0.173	0.275	-0.631	.5283	0.392	1	.5315
Parliamentary cycles[b]	-0.477	0.328	-1.454	.146	2.123	1	.1451

Note. Response variable: first-person personal pronouns (singular = 0, plural = 1).

Significance of a fixed effect was evaluated by model-comparison using likelihood ratio test: a model containing the fixed effect was compared to a model containing both fixed effects.

[a] reference-category: Orbán. [b] reference-category: premiership.

Table S11

GLMM Results for First-Person Verb Conjugations in Orbán and Gyurcsány's Parliamentary Speeches as PMs and as Member of Parliament After Exclusion of Outlier Speech Samples

Fixed effects	β	SE	z	*P*	χ^2	df	*P*
Prime ministers[a]	0.066	0.289	0.227	.8202	0.051	1	.8207
Parliamentary cycles[b]	-0.587	0.321	-1.83	.0672	3.314	1	.0687

Note. Response variable: first-person personal pronouns (singular = 0, plural = 1).

Significance of a fixed effect was evaluated by model-comparison using likelihood ratio test: a model containing the fixed effect was compared to a model containing both fixed effects.

[a] reference-category: Orbán. [b] reference-category: premiership.

Table S12

Frequencies of First-Person Verbal Conjugations in Orbán and Gyurcsány's Parliamentary Speeches During the Parliamentary Cycle Preceding Their First Premiership and During Their First Premiership

	First-person verbal conjugations		
Prime ministers	n	%	n (s.s.)
F. Gyurcsány			
Before (2003-2004)	72	51	7
First (2004 – 2006)	210	30	32
V. Orbán			
Before (1994-1998)	52	42	7
First (1998-2002)	379	32	53

Note. n (s.s.) = number of speech samples. % = percentage of plurals

Table S13

GLMM Results for First-Person Verbal Conjugations in Orbán and Gyurcsány's Parliamentary Speeches During the Parliamentary Cycle Preceding Their First Premiership and During Their First Premiership

Fixed effects	β	SE	z	*P*	χ^2	df	*P*
Prime ministers[a]	0.38	0.256	1.486	.1373	3.313	1	.1283
Parliamentary cycles[b]	-0.853	.312	-2.732	.0063	7.318	1	.0068

Note. Response variable: first-person personal pronouns (singular = 0, plural = 1).

Significance of a fixed effect was evaluated by model-comparison using likelihood ratio test: a model containing the fixed effect was compared to a model containing both fixed effects.

[a] reference-category: Orbán. [b] reference-category: parliamentary cycle preceding first premiership.

Supplementary Figures

Figure S1

Model Diagnostics of GLMMs in the Analyses of WE:I ratio for Personal Pronouns

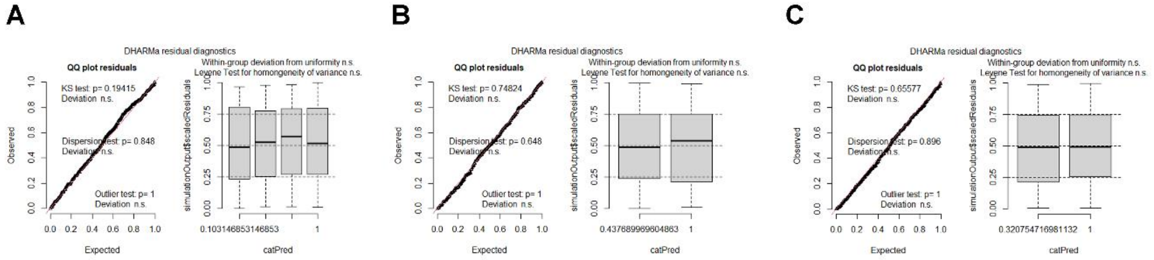

Note. Panel A) Diagnostics of GLMM comparing all four PMs. Panel B) Diagnostics of GLMM comparing Gyurcsány's and Orbán's first and second premiership. Panel C) Diagnostics of GLMM comparing Orbán's second and third premiership.

Figure S2

Model Diagnostics and Distribution of Items in the Comparison of the Four PMs' WE:I Ratio for Verb Conjugations

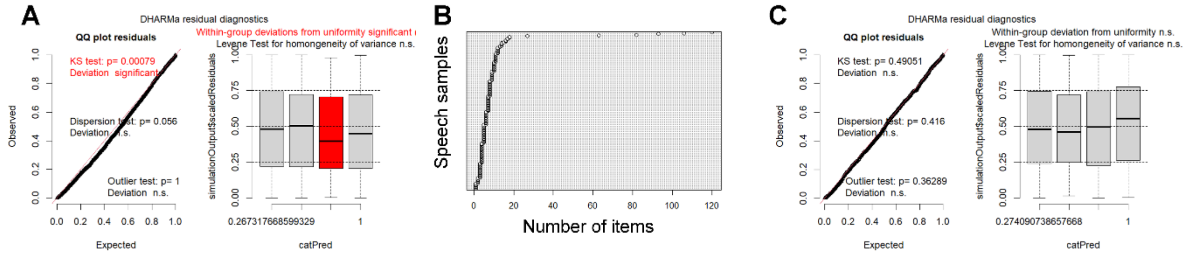

Note. Panel A) Diagnostics of GLMM run on the original dataset. Panel B) Distribution of items (i.e., number of WEs and Is) for each speech sample in the original dataset. Panel C) Diagnostics of GLMM run on the dataset with outlier exclusion.

Figure S3

Model Diagnostics and Distribution of Items in the Comparison of Orbán's and Gyurcsány's WE:I Ratio for Verb Conjugations in Their First and Second Premiership

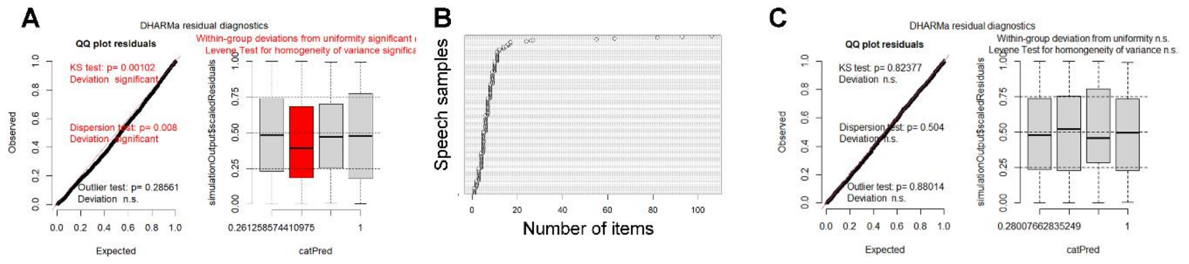

Note. Panel A) Diagnostics of GLMM run on the original dataset. Panel B) Distribution of items (i.e., number of WEs and Is) for each speech sample in the original dataset. Panel C) Diagnostics of GLMM run on the dataset with outlier exclusion.

Figure S4

Model Diagnostics and Distribution of Items in the Comparison of Orbán's WE:I Ratio for Verb Conjugations in his Second and Third Premiership

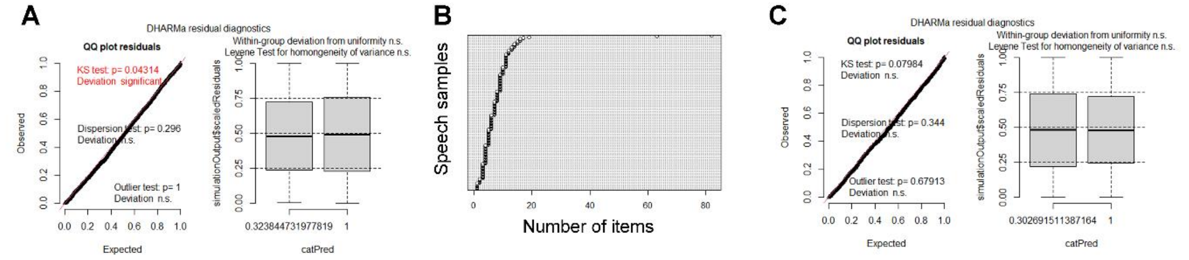

Note. Panel A) Diagnostics of GLMM run on the original dataset. Panel B) Distribution of items (i.e., number of WEs and Is) for each speech sample in the original dataset. Panel C) Diagnostics of GLMM run on the dataset with outlier exclusion.

Figure S5

Model Diagnostics and Distribution of Items in the Comparison of WE:I Ratio for Personal Pronouns of Orbán and Gyurcsány During a Parliamentary Cycle When They were PMs and During a Following Parliamentary Cycle When They Were Simply Members of Parliament

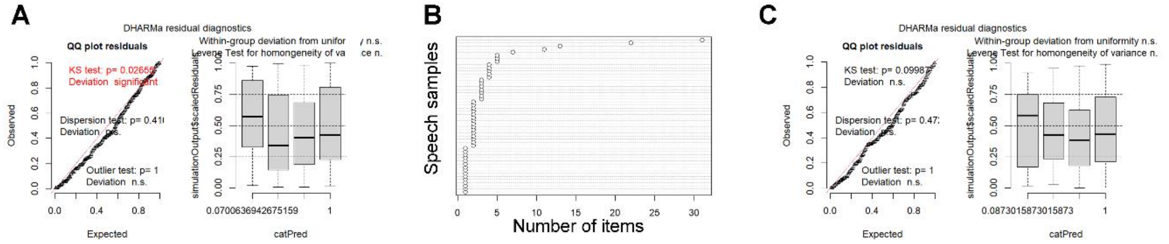

Note. Panel A) Diagnostics of GLMM run on the original dataset. Panel B) Distribution of items (i.e., number of WEs and Is) for each speech sample in the original dataset. Panel C) Diagnostics of GLMM run on the dataset with outlier exclusion.

Figure S6

Model Diagnostics and Distribution of Items in the Comparison of WE:I Ratio for Verb Conjugations of Orbán and Gyurcsány During a Parliamentary Cycle When They Were PMs and During a Following Parliamentary Cycle When They Were Simply Member of Parliament

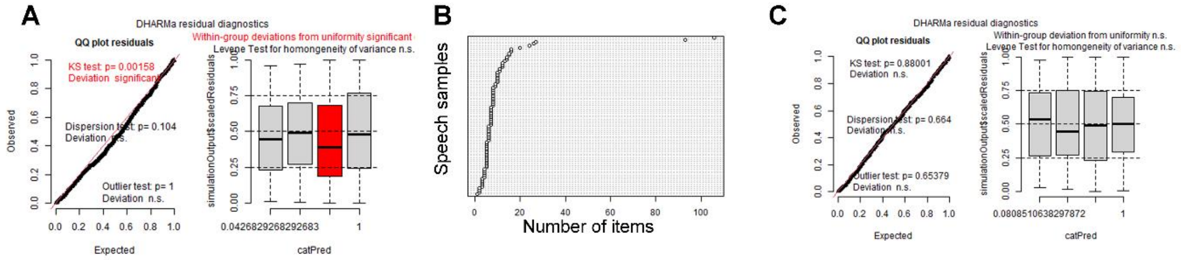

Note. Panel A) Diagnostics of GLMM run on the original dataset. Panel B) Distribution of items (i.e., number of WEs and Is) for each speech sample in the original dataset. Panel C) Diagnostics of GLMM run on the dataset with outlier exclusion.

Figure S7

Model Diagnostics of GLMMs in the Analyses of WE:I Ratio for Verb Conjugations in Speeches During the Parliamentary Cycle Preceding Orbán's and Gyurcsány's First Premiership and During Their First Premiership

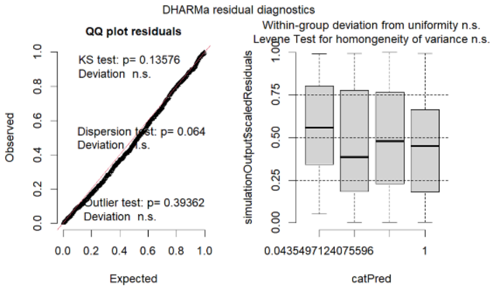

Supplement: S1 File — (PDF) [file pone.0273226.s001.pdf]
